# Anti-Spike Protein Assays to Determine SARS-CoV-2 Antibody Levels: a Head-to-Head Comparison of Five Quantitative Assays

**DOI:** 10.1128/spectrum.00247-21

**Published:** 2021-06-30

**Authors:** Thomas Perkmann, Nicole Perkmann-Nagele, Thomas Koller, Patrick Mucher, Astrid Radakovics, Rodrig Marculescu, Michael Wolzt, Oswald F. Wagner, Christoph J. Binder, Helmuth Haslacher

**Affiliations:** a Department of Laboratory Medicine, Medical University of Vienna, Vienna, Austria; b Department of Clinical Pharmacology, Medical University of Vienna, Vienna, Austria; University of Cincinnati

**Keywords:** comparison, quantitative antibody assays, SARS-CoV-2, vaccination, assay standardization, immunization, quantitative methods, serology

## Abstract

Reliable quantification of the antibody response to severe acute respiratory syndrome coronavirus 2 (SARS-CoV-2) is highly relevant, e.g., for identifying possible vaccine failure and estimating the time of protection. Therefore, we evaluated five different anti-SARS-CoV-2 antibody assays regarding the quantification of anti-spike (S) antibodies. Sera from 69 SARS-CoV-2-naive individuals 21 ± 1 days after vaccination with a single dose of BNT162b2 (Pfizer/BioNTech) were tested using the following quantitative assays: Roche S total antibody, DiaSorin trimeric spike IgG, DiaSorin S1/S2 IgG, Abbott II IgG, and Serion/Virion IgG. Results were further compared to the percent inhibition calculated from a surrogate virus neutralization test (sVNT). Individual values were distributed over several orders of magnitude for all assays. Although the assays were in good overall agreement (ρ = 0.80 to 0.94), Passing-Bablok regression revealed systematic constant and proportional differences, which could not be eliminated by converting the results to binding antibody units (BAU) per milliliter, as suggested by the manufacturers. Seven (10%) individuals had negative sVNT results (i.e., <30% inhibition). These samples were identified by most assays and yielded significantly lower binding antibody levels. Although all assays showed good correlation, they were not interchangeable, even when converted to BAU per milliliter using the WHO international standard for SARS-CoV-2 immunoglobulin. This highlights the need for further standardization of SARS-CoV-2 serology.

**IMPORTANCE** Reliable quantification of the antibody response to SARS-CoV-2 is highly relevant, e.g., for identifying possible vaccine failure and estimating the time of protection. We compared the performance of five CE marked tests that quantify antibodies against the viral spike protein. Our findings suggest that, although all assays showed good correlation, their results were not interchangeable, even when converted to BAU per milliliter using the WHO international standard for SARS-CoV-2 immunoglobulin. This highlights the need for further standardization of SARS-CoV-2 serology.

## INTRODUCTION

Severe acute respiratory syndrome coronavirus 2 (SARS-CoV-2) antibody testing played and still plays an essential role in the management of the coronavirus 2019 (COVID-19) pandemic (1). Detection of specific antibodies following SARS-CoV-2 infection is important at both the individual and population levels to identify those at risk of infection (2). However, now, in the early vaccination era of the COVID-19 pandemic, another essential role of SARS-CoV-2 serology is added: the determination of specific antibodies after active immunization (3, 4).

The first SARS-CoV-2 antibody testing systems were designed to distinguish individuals with prior COVID-19 infection from those who were still naive to this new virus (5). Therefore, these immunoassays were usually developed as qualitative rather than quantitative tests and were designed by the manufacturer to achieve the highest possible specificity and high sensitivity. High specificity was indispensable, especially at the beginning of the pandemic, because the extremely low seroprevalence rates led to many false positives and low positive predictive values even with tests having a specificity of 99% (6). In contrast, the sensitivity of SARS-CoV-2 testing was often reduced to ensure the high specificities needed for these assays (7). The lower antibody levels further aggravated suboptimal sensitivities in mild/asymptomatic infections and during the pandemic by the natural decline in antibody levels (8–13).

Various antigens have been used for this purpose, but essentially two types can be distinguished: nucleocapsid (NC)- and spike protein (S)-based assays (14). Antibodies directed against SARS-CoV-2 specific nucleocapsid antigens are induced early and strongly in most infected individuals due to the virus nucleocapsid's typical strong immunogenicity (15). Furthermore, a very high specificity can be achieved by targeted modification of the nucleocapsid antigen so that no cross-reactivity is observed even with closely related viruses. The discriminatory properties of such nucleocapsid-based antibody assays can therefore be excellent (16, 17). The physiological significance of these antibodies, on the other hand, is unclear, and these surrogate markers for a previous infection are unlikely to be functionally relevant to confer protection or immunity. The antibodies that react with the spike protein, however, act differently. At least a proportion of these S-binding antibodies are likely to have the function of neutralizing antibodies (18). Thus, it is not surprising that numerous studies have shown a correlation between spike protein binding assays and various forms of functional virus neutralization assays (19–24).

In the context of SARS-CoV-2 vaccines, it is precisely these neutralizing antibodies that are of paramount importance. The primary goal of active immunization is to induce many SARS-CoV-2-specific neutralizing antibodies that ideally prevent the pathogen's entry and thus infection or stop the systemic spread to prevent disease (25). The functional virus neutralization assays are not feasible everywhere: assays with live viruses require biosafety level 3, but variants such as pseudotyped neutralization assays are also labor-intensive and cannot be performed at high throughput (26–28). Classical antibody assays, which measure the reactivity of antibodies in serum/plasma with defined antigens, can be performed very rapidly and in high throughput, in contrast to neutralization tests.

Thus, anti-spike protein assays will play an important role in the future. However, these test systems must be able to reliably quantitate SARS-CoV-2-specific antibody levels, be comparable to each other, and have good to excellent agreement with the presence of neutralizing antibodies. The comparability of antibody assays is expected to be improved by the recent introduction of a first WHO international standard for anti-SARS-CoV-2 immunoglobulin (NIBSC code 20/136) with reference to neutralizing antibodies.

In the present work, we aimed to go a step further and compare five commercial quantitative anti-spike protein antibody assays (4 of them with manufacturer's correction factor for the WHO standard) head-to-head in serum samples from 69 individuals who received a single dose of BNT162b (Pfizer/BioNTech).

## RESULTS

### Measurement ranges differ between binding assays.

**FIG 1:**
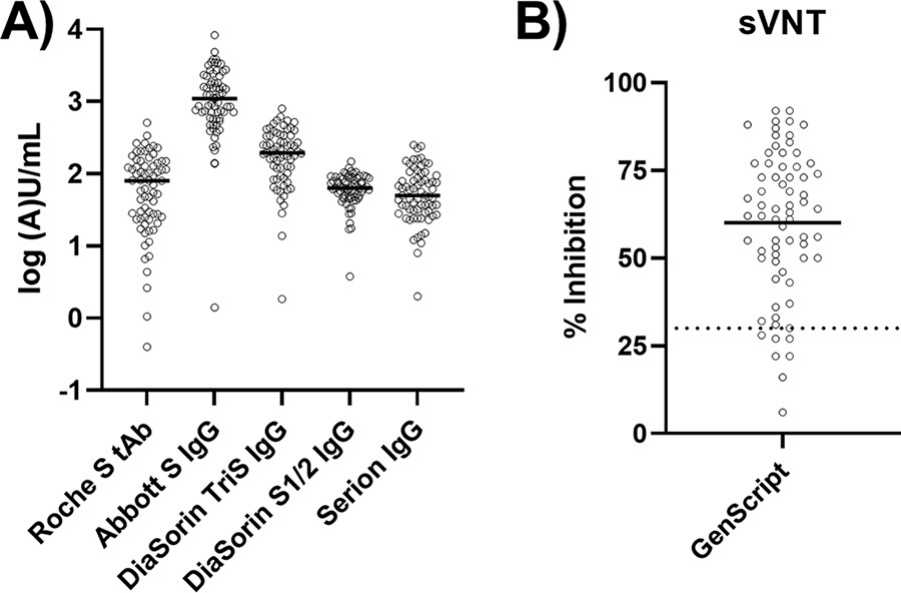
Results from binding assays (A) and sVNTs (B). Solid lines mark the medians. The dotted line (B) marks the manufacturer’s threshold for positivity (30%).

Twenty-nine female (42%) and 40 male (58%) participants with a median age of 42 years (29 to 51) were included. Results from the five different antibody binding assays are presented in Table 1 and Fig. 1. The Abbott S IgG assay showed the highest values with a median of 1,097.1 arbitrary units (AU)/ml (580.1 to 1,959.5 AU/ml) and the broadest range (1.4 to 8,281.0 AU/ml). In contrast, the DiaSorin S1/2 IgG CLIA yielded the lowest values (63.7 AU/ml [47.8 to 87.5 AU/ml]), and the levels ranged from below the limit of quantification (<3.8 AU/ml, 1 sample) to 148.0 AU/ml. Two assays, the DiaSorin Tris IgG (195.0 AU/ml [99.0 to 337.3 AU/ml]) and the Serion IgG (50 U/ml [30 to 89 U/ml]), returned a result above the measuring range for the same donor (>800 AU/ml and >250 U/ml). However, both tests used their full available range (lowest values, 1.8 AU/ml and <3 U/ml). Roche S tAb (total antibody) electrochemiluminescence sandwich immunoassay (ECLIA) results fell between those of the other test systems (79.6 U/ml [24.7-142.3 U/ml]), ranging from 0.4 to 508.0 U/ml.

**TABLE 1:** Measures of position and spread for 5 S-protein based SARS-CoV-2 antibody assays calculated from 69 samples taken 21 ± 1 days after the first shot of BNT162b2

Measure	SARS-CoV-2 antibody test:				
	Roche S tAb	Abbott S IgG	DiaSorin TriS IgG	DiaSorin S1/2 IgG	Serion IgG
Median	79.6	1,097.1	195.0	63.7	50
5th percentile	4.3	207.5	36.4	20.5	12
25th percentile	24.7	580.1	99.0	47.8	30
75th percentile	142.3	1,959.5	337.3	87.5	89
95th percentile	265	3,812.4	545.3	105.2	169
Mean	96.4	1,494.2	230.4	66.3	69
SD	92.9	1,367.2	163.2	26.5	55
Range	0.4 to 508.0	1.4 to 8,281.0	1.8 to >800.0	<3.8 to 148.0	2 to >250

The measured values indicate that the numerical results are strongly dependent on the test system used. In the next step, we aimed to evaluate the overall agreement between the test systems.

### Agreement between results from different binding assays.

Results from the Roche S tAb assay correlated well with those of the other binding assays (Abbott S IgG ρ = 0.88, DiaSorin TriS IgG ρ = 0.83, DiaSorin S1/2 IgG ρ = 0.80, Serion IgG ρ = 0.82). However, Passing-Bablok regression revealed relevant systematic proportional and constant differences: Abbott S IgG = 82.5 + 15.54*x*, DiaSorin TriS IgG = 33.4 + 2.18*x*, DiaSorin S1/2 IgG = 39.6 + 0.32*x*, Serion IgG = 12.3 + 0.65*x*.

The Abbott S IgG assay correlated at a ρ value of 0.90 with the remaining three test systems (DiaSorin TriS IgG and S1/2 IgG and Serion IgG). In Passing-Bablok regression, all constant and proportional errors were statistically significant: DiaSorin TriS IgG = 24.5 + 0.13*x*, DiaSorin S1/2 IgG = 34.5 + 0.02*x*, Serion IgG = 6.2 + 0.04*x*.

The DiaSorin TriS IgG assay showed an excellent correlation with the remaining two tests (DiaSorin S1/2 IgG ρ = 0.91, Serion IgG ρ = 0.94). In the Passing-Bablok regression, nevertheless, marked deviations became apparent: DiaSorin S1/2 IgG = 30.5 + 0.16*x*, Serion IgG = −0.0 + 0.31*x*.

**FIG 2:**
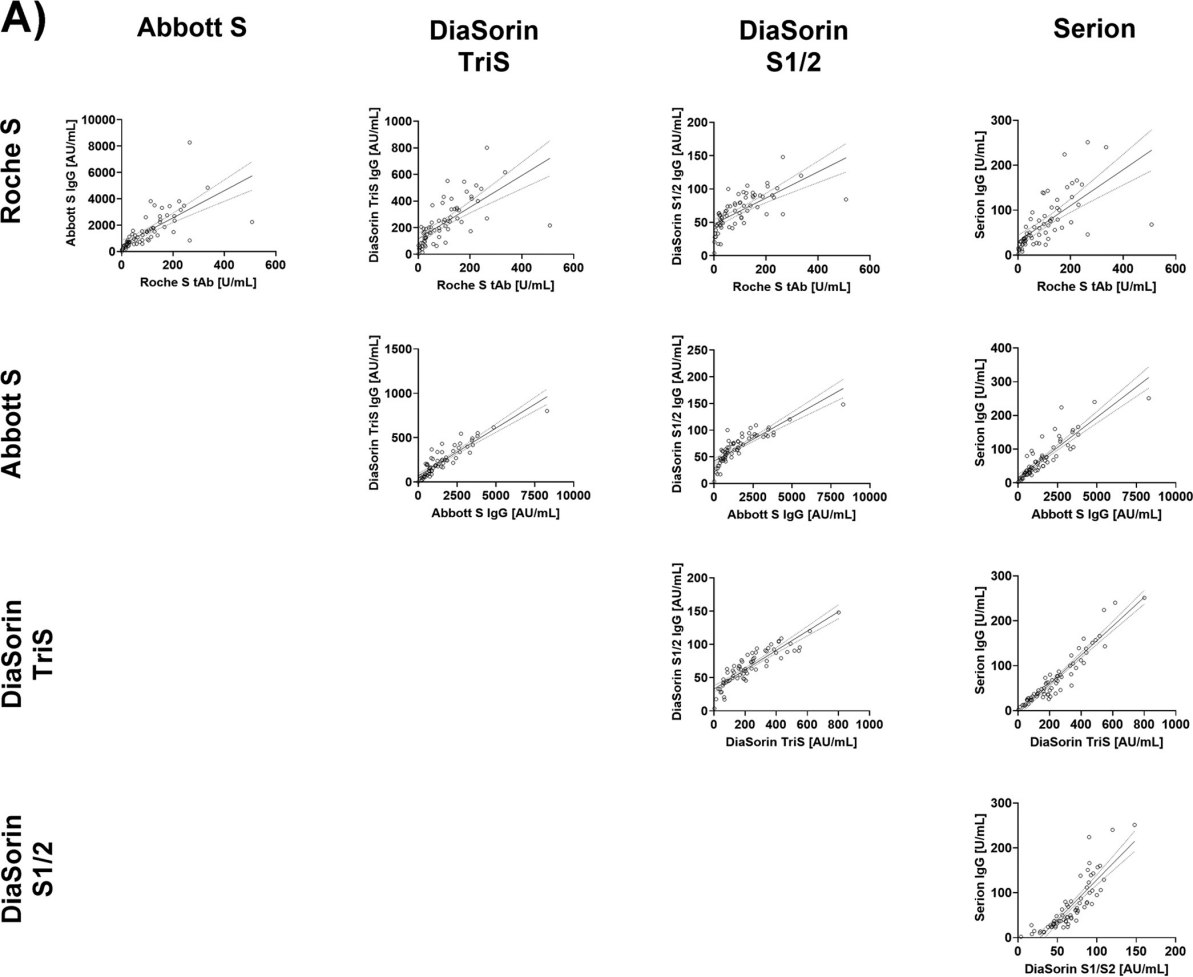
Comparison of binding assays by linear regression (dotted lines indicate the 95% confidence interval) (A) and residual plots (B).

Finally, the DiaSorin S1/2 IgG and the Serion IgG correlated at a ρ value of 0.91, and the Passing-Bablok regression equation was Serion IgG = −50.9 + 1.78*x*. All described relationships, as well as related residual plots, are presented in Fig. 2.

Furthermore, we assessed whether the classification of results into tertiles (0 to 33.3%, 33.4 to 66.7%, and 66.8 to 100%) was comparable, e.g., whether a sample yielding a result in the lowest tertile of test A was also in the lowest tertile of test B. Cohen’s kappa was between 0.60 and 0.80, indicating a good agreement, for all but for one of the 10 test combinations (Roche S tAb/Serion, kappa = 0.59 [Table 2]).

**TABLE 2:** Kappa values for 10 different test combinations regarding the classifications of samples into tertiles

Test	Kappa ± 95% confidence interval			
	Abbott S IgG	DiaSorin TriS IgG	DiaSorin S1/2 IgG	Serion IgG
Roche S tAb	0.72 ± 0.06	0.69 ± 0.06	0.63 ± 0.07	0.59 ± 0.07
Abbott S IgG		0.64 ± 0.07	0.74 ± 0.06	0.64 ± 0.07
DiaSorin TriS IgG			0.74 ± 0.06	0.74 ± 0.06
DiaSorin S1/2 IgG				0.80 ± 0.05

In conclusion, the results of the investigated test systems correlate well but are not necessarily interchangeable. Several manufacturers provided conversion factors related to the WHO international standard for SARS-CoV-2 immunoglobulin, as described in Materials and Methods. Next, we wanted to clarify whether comparing values converted to binding antibody units (BAU) per milliliter instead of arbitrary values facilitates comparability.

### Associations between standardized binding assay results.

The numbers of BAU per milliliter were calculated for the Abbott S IgG, the DiaSorin TriS IgG, and the Serion IgG, according to the recently proposed conversion factors. Results from the Roche S tAb ECLIA did not require conversion, as indicated by the manufacturer.

**FIG 3:**
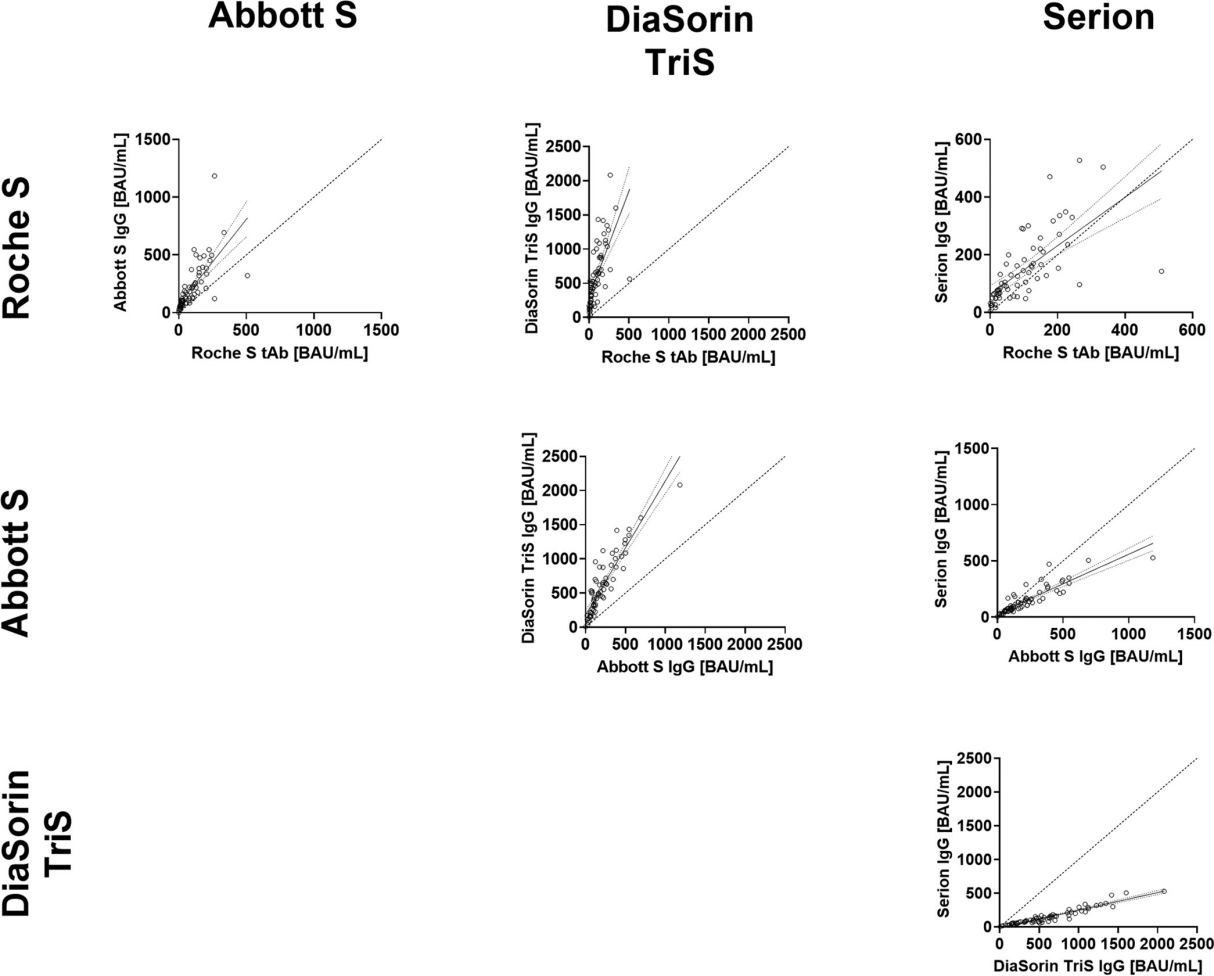
Comparison of binding assay results converted to BAU per milliliter. Given are linear regression curves and their 95% confidence intervals. Dotted diagonal lines represent lines of equality.

As shown in Fig. 3, the recalculation of BAU per milliliter did not solve the problem of high proportional errors. The least proportional error could be observed for the relationship between Roche S tAb and Serion IgG. However, the same combination was characterized by comparatively high variability (ρ = 0.82).

### Correlation of binding assay results with a surrogate neutralization assay.

**FIG 4:**
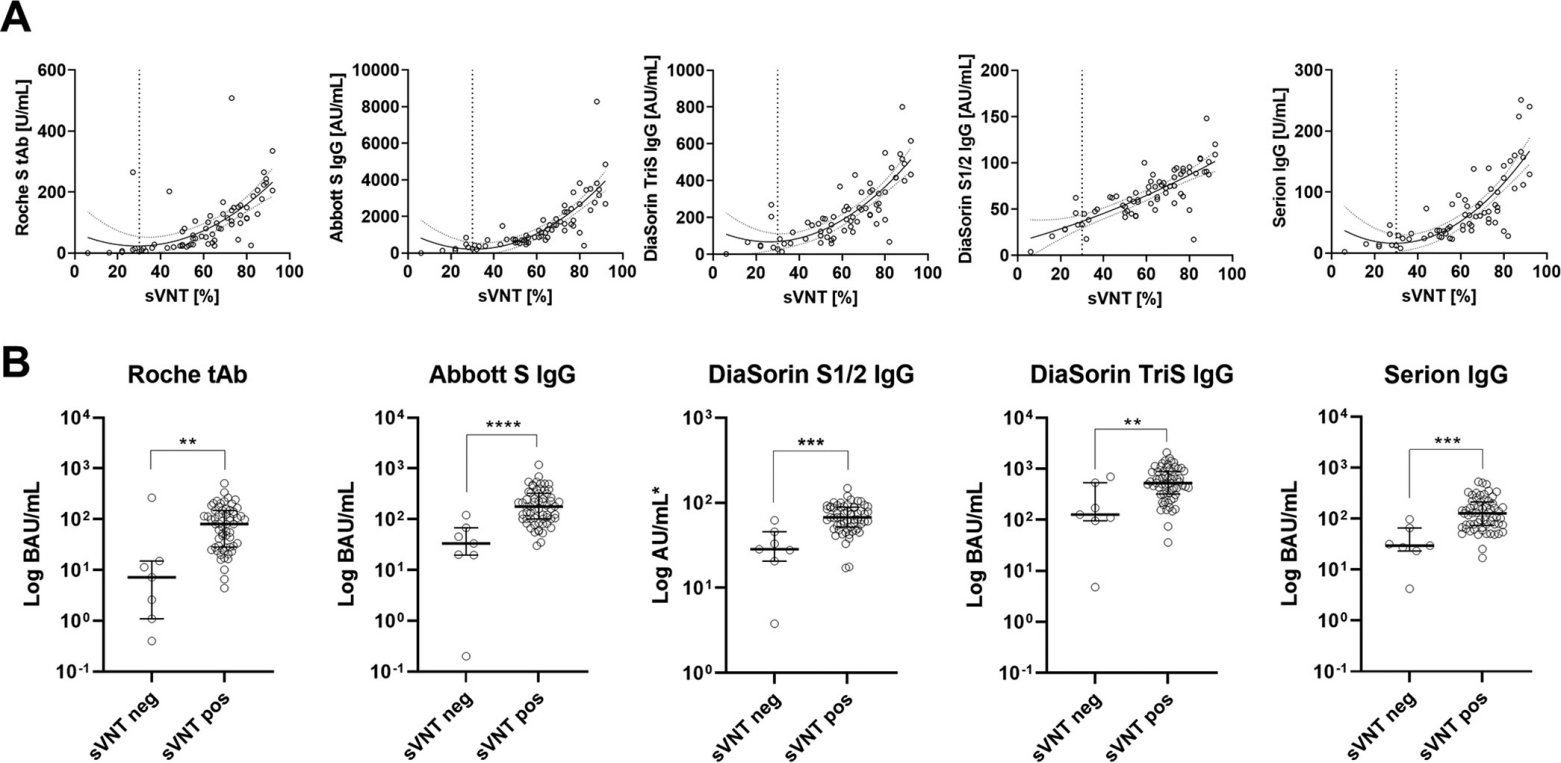
(A) Relationships between binding assay results and percent inhibition assayed using a surrogate virus neutralization test (threshold for positivity, 30% [dotted vertical lines]). Presented are quadratic regression lines and their 95% confidence intervals. (B) Comparison (Mann-Whitney tests) of standardized (BAU per milliliter) binding antibody levels (except for DiaSorin S1/2 IgG, for which a conversion factor to BAU per milliliter is not available) between samples with a positive (sVNT pos) or negative (sVNT neg) surrogate virus neutralization test result. **, *P* < 0.01; ***, *P* < 0.001; ****, *P* < 0.0001.

In a final step, the binding assays’ results were compared to percent inhibition of a surrogate virus neutralization test (sVNT). In the sVNT, the tested samples yielded median values of 63% (50 to 76%), ranging from 6% to 92%. Figure 4A illustrates that all binding assays except the DiaSorin S1/2 IgG showed a quadratic relationship with the sVNT. The binding assays also differentiated those values clustered in the upper range of the sVNT. However, for the DiaSorin S1/2, the quadratic curve approached a straight line, indicating a mostly linear relationship between this binding assay and the sVNT within the observed range.

Seven (10%) of the individuals yielded sVNT results below 30% inhibition, which is considered negative according to the manufacturer (Fig. 1). Binding assay results were compared between positives and negatives in the sVNT by Mann-Whitney tests. The median (interquartile range [IQR]) binding assay results for sVNT-negative and -positive samples were as follows: Roche tAb, 7.2 U/ml (1.4 to 14.1 U/ml) and 80.9 U/ml (28.4 to 149.0 U/ml); Abbott S IgG, 233.8 AU/ml (138.8 to 436.0 AU/ml) and 1,234.3 AU/ml (710.2 to 2,246.9 AU/ml); DiaSorin S1/2 IgG, 28.4 AU/ml (22.4 to 42.5 AU/ml) and 67.5 AU/ml (52.4 to 88.6 AU/ml); DiaSorin TriS IgG, 48.8 AU/ml (38.3 to 170.1 AU/ml) and 202.0 AU/ml (124.0 to 339.0 AU/ml); and Serion IgG, 14 U/ml (12 to 27 U/ml) and 61 U/ml (36 to 100 U/ml). Values recalculated to BAU per milliliter, if applicable, are given in Fig. 4B.

## DISCUSSION

SARS-CoV-2 antibody assays become important tools to evaluate the proportion of people affected by COVID-19 and identify those who are still at infection risk. Now, with the first vaccines available, a new field of use for SARS-CoV-2 antibody tests will open up. First, many vaccinated individuals will be interested in confirming their own vaccination success based on the detection of specific antibodies. Second, vaccination-induced antibodies may be used as surrogate from which a protection correlate will be estimated. To date, only limited information on the performance of quantitative SARS-CoV-2 antibody assays is available, since most currently evaluated assays were developed in-house, as recently summarized by the CDC COVID-19 response group (29). Only for a few commercially available quantitative CE-marked test systems are preliminary data on the performance given in the literature (17, 20, 30–32).

Although a protection correlate for immunity in SARS-CoV-2 has not been defined yet, it is useful to begin this important preliminary work now (33). Therefore, we compared different commercial SARS-CoV-2 antibody assays with spike protein reactivity using a vaccination cohort to give a first insight into the comparability of these assays.

With regard to the numerical results, we were able to determine a broad distribution of values for each individual test system, so that these were presented on a logarithmic scale. This is in line with recently published reports, showing the antibody response after a single dose of BNT162b2 vaccine (3, 4). Interestingly, in agreement with a study involving >500 participants in an identical study setting, we observed very similar mean values for the measurements with the DiaSorin S1/S2 IgG: 66.3 AU/ml versus 68.6 AU/ml (3). Therefore, it is reasonable to assume that our cohort is representative despite the moderate number of participants. In addition, we were able to show that the results of the different test systems varied by a factor of up to more than 50. This leads to the initial conclusion that a direct comparability of the numerical results of different test systems is unlikely to be given across the range of individual findings. Differences also occurred with respect to measurement ranges, and upper measurement limits were exceeded in 2 of 5 systems (DiaSorin TriS IgG and Serion IgG), although the study cohort reflects the antibody response before the administration of the second dose of the Pfizer/BioNTech vaccine in SARS-CoV-2 naive individuals. However, it must be mentioned that it is not yet known up to what level a differentiation of the obtained values is meaningful. Nevertheless, it can be assumed that the average values of completely vaccinated persons are significantly higher than those in our collective, and thus, the upper measurement limits could frequently be exceeded in most assays. If clinically relevant, this could make additional dilution steps necessary, which are not yet taken into account by the manufacturers.

Despite the different levels of measurement, all systems showed good correlations with each other. When the measured values of the individual antibody tests were assigned to tertiles, good agreement was shown between the lowest third, the middle third, and the highest third of the results. Thus, one individual with known immunosuppressive therapy consistently showed no formation of antibodies in all five antibody binding assays tested. With defined cutoffs for low or high vaccination titers of the different test systems, at least a partial transferability of a result from one to another test system may therefore be expected.

Such transferability of results could also be anticipated via referencing the antibody assays used to an international reference standard (29). Indeed, a first WHO international SARS-CoV-2 antibody standard with the valence of 1,000 BAU/ml has recently become available. This standard was used by the manufacturers for four of the five assays studied. However, this standardization was introduced not during the establishment of the test system but *post hoc* as a reference to define a conversion factor of their own units in BAU per milliliter. It is therefore not surprising that this subsequent correction did not reduce the existing systematic deviations (Fig. 2) between the different tests. Only the Roche S tAb and Serion IgG tests were able to approximate the equivalence line, although here a very wide scattering of values around the trend lines was observed.

The *in vitro* binding of infection-associated antibodies to pathogen-specific antigens in an antibody test are important markers to objectify a past infection or vaccination. However, these do not necessarily say anything about the function of these antibodies (1). Only those antibodies that will prevent the virus from binding to the cellular receptor, the ACE2 receptor, via the surface spike protein (34, 35), act as neutralizing antibodies. Tests to neutralize live viruses can only be performed in very specialized laboratories and unfortunately, in the case of SARS-CoV-2, are not standardized, making comparability almost impossible. For this reason, we chose to use a well-characterized surrogate virus neutralization test (sVNT) as a functional reference (36–38). In this assay, a simple enzyme-linked immunosorbent assay (ELISA) format is used to determine the inhibition of conjugated receptor-binding domain (RBD) protein by neutralizing antibodies to the plate-bound ACE2 receptor. The manufacturer suggests a threshold for positivity of 30% inhibition. With the exception of the Serion IgG assay, where the median of samples with negative sVNT results was borderline (14 U/ml), the medians of sVNT-negative samples were above the thresholds for positivity in all other test systems. This implies that the cutoff values given for the respective test systems are only valid for the diagnosis of a past infection and do not necessarily represent a threshold value for the presence of sufficient neutralizing activity.

In conclusion, we found good correlation between all evaluated assays; however, the values from the different test systems were not interchangeable, even when converted to BAU per milliliter using the WHO international standard for SARS-CoV-2 immunoglobulin. Furthermore, it should be noted that the thresholds for positivity provided by the manufacturers are of diagnostic value and are not indicative of sufficient inhibitory capacities.

## MATERIALS AND METHODS

### Study design and participants.

This prospective observational study was performed using sera collected in February 2021 from 69 individuals without a previous SARS-CoV-2 infection in the course of a workplace vaccination campaign in the metropolitan area of Vienna, Austria. The samples were taken 21 ± 1 days (mean ± standard deviation) after the first dose of the Pfizer/BioNTech BNT162b2 vaccine. We included vaccinated persons rather than individuals with a history of SARS-CoV-2 infection, in order to be able to compare test systems following a more or less standardized stimulus. Further inclusion criteria were an age of >18 years, whereas an insufficient amount of serum resulted in exclusion from the study. The study protocol was reviewed and approved by the Ethics Committee of the Medical University of Vienna (EK1066/2021). All participants provided written informed consent to donate blood for the evaluation of diagnostic test systems (EK404/2012). The studied complied with the World Medical Association Declaration of Helsinki regarding ethical conduct of research involving human subjects.

### Laboratory procedures.

Serum was obtained and stored at 2 to 10°C for <7 days within the MedUni Wien Biobank, a centralized facility for the preparation and storage of biomaterial with certified quality management (ISO 9001:2015) (39). All analytical procedures were performed at the Department for Laboratory Medicine, Medical University of Vienna. The following CE-marked binding assays were applied:

The Roche Elecsys anti-SARS-CoV-2 S (Roche S tAb) is an electrochemiluminescence sandwich immunoassay (ECLIA) and detects total antibodies directed against the receptor-binding domain (RBD) of the viral spike (S) protein. It was measured on cobas e801 modular analyzers (Roche Diagnostics, Rotkreuz, Switzerland). The quantification range is between 0.4 and 2,500.0 U/ml, and 0.8 U/ml is used as a cutoff for positivity.

The Abbott SARS-CoV-2 IgG II Quant-test (Abbott S IgG) is a chemiluminescence microparticle immunoassay (CMIA). It quantifies IgG-type antibodies against the RBD of the viral S-protein on an Abbott Architect platform (Abbott, Abbott Park, IL, USA) between 21.0 and 40,000.0 AU/ml, with ≥50 AU/ml as a threshold for positivity.

The DiaSorin Liaison SARS-CoV-2 TrimericS IgG (DiaSorin TriS IgG) chemiluminescence immunoassay (CLIA) quantifies IgG antibodies against a trimeric S-protein antigen on a DiaSorin Liaison (DiaSorin, Stillwater, OK, USA). The quantification range is between 1.63 and 800 AU/ml. Samples with values of ≥13 AU/ml are considered positive.

The DiaSorin Liaison SARS-CoV-2 S1/2 CLIA (DiaSorin S1/2 IgG) detects IgG antibodies against an S1/S2 combination antigen on a DiaSorin Liaison (DiaSorin, Stillwater, OK, USA). The quantification range is between 3.8 and 400.0 AU/ml. The cutoff for positivity is >15 AU/ml, whereby results between 12.0 and 15.0 AU/ml are considered borderline.

The Virion\Serion ELISA (enzyme-linked immunosorbent assay) agile SARS-CoV-2 IgG assay (Serion IgG) (Institut Virion-Serion, Wuerzburg, Germany) was analyzed on a FilterMax F5 multiplate reader (Molecular Devices, San Jose, CA, USA) and quantifies IgG antibodies against total S protein between 3 and 250 U/ml. The threshold for positivity is 15 U/ml, with values between 10 and 15 U/ml being considered borderline results.

Binding antibody units (BAU) per milliliter, which are traceable to the WHO international standard for anti-SARS-CoV-2 immunoglobulin, were calculated by applying the following conversion factors, as suggested by the manufacturers: Roche S tAb, (U/ml) × 1; Abbott S IgG, (U/ml) × (1/7); DiaSorin TriS IgG, (AU/ml) × 2.6; Serion IgG, (U/ml) × 2.1.

We excluded prior SARS-CoV-2 infection by using the Roche Elecsys SARS-CoV-2 ECLIA on the cobas e801 analyzer (Roche), which detects total antibodies to the viral nucleocapsid antigen. These antibodies are not induced by vaccination with BNT162b2. This assay yields high diagnostic sensitivity (90%) and specificity (99.7%) for infections that occurred at least 14 days before blood withdrawal (5). As suggested by the manufacturer, results of a cutoff index (COI) of >1.000 were considered positive.

Neutralizing capacity was estimated by performing a surrogate virus neutralization test (sVNT) (GenScript, Piscataway, NJ, USA). The assay was read on a FilterMax F5 multimodal plate reader. According to the manufacturer, it shows excellent positive (100% [87.1 to 100.0%]) and negative (100.0% [95.8 to 100.0%]) agreement with conventional plaque reduction neutralization tests (PRNT_50_ and PRNT_90_) and has therefore already been used in various studies (36, 40, 41). Results of ≥30% are considered positive.

### Statistical analysis.

Continuous data are given as medians and interquartile ranges; categorical data are given as counts and percentages. Test systems were compared by Passing-Bablok regressions. This method reveals differences between two test systems by estimating the slope (systematic proportional differences) and the intercept (systematic constant differences) of a linear regression line. The advantage of this well-established method over conventional linear regressions is that no preconditions regarding the distribution of the measured values and the measurement errors have to be met (42). Besides Passing-Bablok regressions, Cohen’s kappa (linear weights) and Spearman rank correlations were applied to evaluate the agreement between binding assays. Kappa is a measure for assessing the compliance between two ratings. In detail, we evaluated the degree to which the different test systems agreed to classify the test samples into tertiles (43). Spearman rank correlation is a method to describe relationships between two variables that do not have to be linear. The relationship between binding assays and results from the sVNT was described by quadratic curve fitting. Statistical significance was assumed if *P* values were below 0.05. All analyses were performed using MedCalc 19.6 (MedCalc, Ostend, Belgium), and graphs were drawn using GraphPad 9 (GraphPad, La Jolla, CA, USA). The underlying data can be requested by interested researchers from the corresponding author.
